# The Effect of Plant-Based Protein Preparations on Quality and Functional Properties of Cream Filling

**DOI:** 10.3390/molecules31101565

**Published:** 2026-05-08

**Authors:** Joanna Miedzianka, Krzysztof Podsiadły, Agnieszka Nemś, Katarzyna Piwowar-Sulej, Agnieszka Kita

**Affiliations:** 1 Department of Food Storage and Technology, Faculty of Biotechnology and Food Science, Wrocław University of Environmental and Life Sciences, 37 Chełmońskiego Street, 51-630 Wroclaw, Poland; agnieszka.nems@upwr.edu.pl (A.N.); agnieszka.kita@upwr.edu.pl (A.K.); 2 Department of Labour, Capital and Innovation, Faculty of Management, Wroclaw University of Economics and Business, 118/120 Komandorska Street, 53-345 Wrocław, Poland; krzysztof.podsiadly@gmail.com (K.P.); katarzyna.piwowar-sulej@ue.wroc.pl (K.P.-S.)

**Keywords:** cream fillings, plant-based protein preparation, physicochemical properties, functional properties, sensory evaluation

## Abstract

Cream fillings are widely used semi-solid components in confectionery products, where the fat phase strongly influences the structure, stability, and sensory characteristics. Increasing consumer demand for plant-based and nutritionally improved foods has stimulated efforts to reduce the palm oil content and incorporate plant-derived proteins; however, systematic studies comparing the functional impact of different commercial protein preparations in such systems remain limited. Therefore, this study investigated the influence of pea, brown rice, pumpkin, hemp, and sunflower protein preparations on the physicochemical, functional, and sensory properties of cream fillings formulated with reduced palm fat. Commercial protein preparations (2.5% and 5%) were incorporated as partial fat replacers, and the chemical composition, amino acid profile, color parameters, water activity, viscosity, and particle size distribution were evaluated. Additionally, multivariate statistical approaches were applied to better understand relationships. The results showed that pea protein concentrate improved the nutritional profile and provided the most favorable balance of texture and sensory attributes. Other proteins also modified the physical properties of the fillings, although to different extents, while increasing the protein concentration generally intensified color changes and increased viscosity. Overall, the findings provide new insights into the behavior of commercial plant protein preparations in fat-rich confectionery systems and support the development of plant-enriched cream fillings with a reduced palm oil content.

## 1. Introduction

Cream fillings are widely used semi-solid components in confectionery products, such as biscuits, wafers, and sandwich cookies. Their technological and sensory properties are largely determined by the fat phase, which contributes to the texture, mouthfeel, and structural stability of the product. Palm oil is commonly used in confectionery fillings due to its favorable melting profile, oxidative stability, and ability to form stable fat crystal networks that provide the desired creamy texture and spreadability. This oil is one of the most widely used edible fats worldwide and is important in confectionery technology due to its unique physicochemical characteristics [1]. However, the high content of saturated fatty acids in palm oil has raised nutritional concerns, as diets high in saturated fats are associated with an increased risk of chronic diseases [2]. However, beyond nutritional aspects, the extensive use of palm oil is also associated with significant environmental concerns. The large-scale cultivation of oil palm contributes to deforestation, the loss of biodiversity, and increased greenhouse gas emissions, thereby raising important sustainability issues in the context of global food production. These concerns have stimulated interest in the development of reduced-fat or nutritionally improved food formulations that maintain desirable technological and sensory qualities. Moreover, the excessive consumption of fat- and sugar-rich confectionery products is associated with an increased risk of diet-related diseases, including obesity and cardiovascular disorders. In particular, the presence of trans fatty acids, formed during the hydrogenation of vegetable oils, has been linked to adverse health effects. In this context, there is a growing interest in alternative ingredients that can partially replace fat while maintaining desirable product properties.

In recent years, plant-based proteins have gained considerable attention as multifunctional food ingredients capable of improving both the nutritional quality and technological properties of processed foods. Plant proteins display functional properties such as water binding, emulsification, gel formation, and viscosity enhancement, which make them attractive candidates for incorporation into reduced-fat food systems. Consumers’ increasing demand for sustainable and plant-based food products has further accelerated research on the application of plant proteins in innovative formulations [3]. This trend reflects a broader shift toward the development of food products that combine improved nutritional profiles with a reduced environmental impact, aligning food innovation with the principles of sustainable development.

Recent studies have demonstrated that plant proteins may act as fat mimetics by forming structured aggregates, microgels, or microparticles that modify the rheological and lubrication properties of food matrices and can contribute to the perception of creaminess in reduced-fat systems. Such fat-mimetic behavior has been attributed to the formation of deformable protein particles that act as lubricating elements during oral processing (“ball-bearing effect”), thereby reducing friction between oral surfaces and mimicking the lubricating properties of fat [4].

Among plant protein sources, preparations derived from legumes, oilseeds, and cereal grains are increasingly used in food formulations due to their favorable amino acid composition and functional properties. Pea protein, for example, exhibits a relatively neutral flavor, good emulsifying capacity, and high nutritional value [5]. Other proteins obtained from oilseed and cereal crops such as pumpkin seeds, hemp seeds, sunflower seeds, or brown rice are increasingly explored as potential functional ingredients due to their distinct compositions and techno-functional behaviors [3]. These differences in origin and composition may translate into distinct functional behavior in complex food matrices. It should also be noted that protein content and composition are strongly influenced by the cultivar, environmental conditions, and agronomic practices. Factors such as the soil quality, temperature, water availability, and fertilization regimes may significantly affect not only the protein yield but also the amino acid profile and the relative proportion of storage proteins. These variations may, in turn, influence key functional properties of protein preparations, including the solubility, emulsifying capacity, and aggregation behavior. As a result, variability related to the raw material origin represents an important challenge for ingredient standardization and industrial applications, particularly in the context of designing functional food systems. In this context, plant proteins are not only considered valuable nutritional components but also sustainable alternatives to animal-derived ingredients, contributing to the development of more environmentally friendly food systems.

Despite the growing interest in plant-protein-based ingredients, their technological performance in complex fat-rich confectionery matrices remains insufficiently examined. This is particularly relevant in the context of current efforts to design food systems that address both health-related and environmental challenges. Cream fillings present a particularly challenging system because their texture and stability depend strongly on the structure of the fat network and interactions between fat crystals and dispersed solid particles. The incorporation of plant protein preparations in such systems may influence not only nutritional value but also the rheological behavior, particle size distribution, color, and sensory characteristics.

Although numerous studies have investigated the application of plant proteins in dairy alternatives, emulsions, and meat analogues, comparative research evaluating different commercial plant protein preparations, specifically in confectionery cream fillings, is still limited. In particular, there is a lack of systematic investigations addressing how various plant protein sources affect the physicochemical and sensory properties of reduced-fat cream fillings, including emulsification, lubrication, and mouthfeel.

Therefore, the aim of this study was to evaluate the effect of incorporating different commercial plant-based protein preparations, including pea, pumpkin, hemp, brown rice, and sunflower proteins, into cream filling formulations as partial palm fat replacers. The study focused on physicochemical, functional, and sensory properties of the obtained cream fillings.

The results may contribute to the development of nutritionally improved confectionery products enriched with plant proteins and containing reduced amounts of palm fat, while also addressing current challenges related to public health and environmental sustainability. It was hypothesized that protein preparations differing in botanical origin and functional properties would exhibit distinct fat-mimetic behavior in cream fillings, leading to measurable differences in viscosity, particle size distribution, and sensory perception. These effects may be mechanistically linked to protein denaturation and aggregation phenomena occurring during industrial processing of protein preparations, which determine the exposure of hydrophobic domains, protein flexibility, and aggregation behavior, ultimately governing interfacial activity, fat-binding capacity, and the resulting rheological and sensory properties of cream fillings. Denaturation exposes hydrophobic regions, promoting protein–protein and protein–lipid interactions, which may lead to aggregate formation and altered functionality in fat-rich systems. Moreover, the presence of non-protein components, such as residual lipids or phenolic compounds, may further modulate interfacial behavior and aggregation patterns. Such an approach enables a systematic understanding of how the plant protein composition and structure translate into macroscopic quality attributes in complex confectionery systems.

## 2. Results and Discussion

The cream fillings containing plant-based protein preparations differed in terms of their chemical properties, amino acid composition, physical properties, and sensory evaluation, compared to the control sample. This study highlights the importance of carefully characterizing the physicochemical and functional performance of commercial plant protein ingredients from different sources in cream fillings. Observed differences may have resulted from protein denaturation, protein aggregation, or the presence of non-protein components that influenced protein performance during commercial processing [6], which together determine the relationship among the protein composition, structural organization, and their functional and sensory performance in complex fat-rich systems. However, the extent and nature of these structural changes strongly depended on the protein source. Legume proteins (e.g., pea) showed a higher tendency to form hydrophobic-driven aggregates, whereas cereal-derived proteins (e.g., brown rice) exhibited more compact and less interactive structures, and seed proteins rich in non-protein fractions (e.g., hemp and pumpkin) were additionally influenced by fiber and lipid residues, which modified interfacial behavior in a different manner for each system. In particular, variations in the amino acid composition, especially in the proportion of hydrophobic residues, may directly affect the interfacial activity, fat-binding capacity, and consequently the perception of creaminess and texture. These effects can be mechanistically interpreted in terms of structural modifications occurring during protein processing. In particular, the partial denaturation of proteins may expose hydrophobic regions, enhancing protein–protein and protein–lipid interactions. This promotes the formation of aggregates with altered interfacial activity, which directly affects the emulsification behavior and fat immobilization in the system. Furthermore, the presence of non-protein components, such as residual lipids, fiber, or phenolic compounds, may interfere with protein adsorption at the fat–water interface, thereby modifying the stability and rheological properties of the cream fillings.

### 2.1. Characteristics of Plant-Based Protein Preparations

The functional and chemical properties of plant-based protein preparations are key factors determining their performance in food systems. The analyzed preparations differed significantly in their chemical composition (Table 1). Among all samples, the highest protein content was found in brown rice (77.85 g/100 g) and pea (74.90 g/100 g) preparations, while the lowest was observed for hemp protein (45.58 g/100 g). However, it is worth emphasizing that the protein content of these seeds depends on different factors and changes with every harvest [7]. Additionally, in all the analyzed samples, the storage proteins dominate, which makes them useful for human consumption. The fat content varied considerably, ranging from 5.81 g/100 g (brown rice) to 12.87 g/100 g (pumpkin), which may influence their functionality in emulsion-based systems, as a higher residual lipid content may promote protein–lipid interactions but also interfere with interfacial adsorption, leading to differences in emulsion stability and sensory perception between protein sources. In particular, pea protein, characterized by a higher proportion of hydrophobic amino acids, is more prone to lipid association and interfacial adsorption, whereas hemp and pumpkin proteins, containing higher levels of non-protein components such as fiber and residual lipids, may interfere with protein film formation at the oil–water interface. Brown rice protein, with a relatively more balanced composition, showed intermediate behavior in this regard. Beyond compositional differences, the functionality of these protein preparations may also depend on their structural state. These structural states, however, were not uniform across protein sources. Pea protein is typically more susceptible to partial unfolding and aggregation, enhancing interfacial activity, whereas rice proteins tend to maintain more compact structures. In contrast, hemp and pumpkin proteins may form heterogeneous aggregates due to the presence of non-protein matrix components, resulting in different functional outcomes in fat-rich systems. Proteins obtained through industrial processing (e.g., defatting, drying, milling) are often partially denatured, which can significantly alter their solubility, aggregation behavior, and interfacial properties. These structural changes may explain differences in oil-binding capacity and performance in fat-rich systems observed in this study.

The amino acid compositions of the protein preparations also showed notable differences (Table 2), which may have direct implications for their structural behavior and functional performance, particularly in terms of nonpolar interactions, protein aggregation, and interfacial activity in fat-rich systems. The total amino acid content ranged from 82.04 mg/g (pumpkin) to 127.14 mg/g (pea). In all analyzed samples, Glu was the dominant amino acid, with values between 14.44 and 20.70 mg/g. Additionally, high levels of Asp, Arg, and Leu were observed, confirming the good nutritional potential of these protein sources.

From a nutritional perspective, the limiting amino acid in most preparations was Lys, except for pea protein, where sulfur-containing amino acids (Met + Cys) were limiting (Table 2). Lysine is commonly deficient in cereal-based proteins, whereas legume proteins, such as pea, are rich in this amino acid, which makes them particularly valuable in improving the overall amino acid balance of plant-based formulations. The presence of only one limiting amino acid in pea, hemp, and brown rice proteins indicates a relatively high biological value compared to pumpkin and sunflower proteins, which were additionally limited by Thr. These observations are consistent with literature data [6,8].

Functional properties, such as the oil-binding capacity (OBC), also differed significantly among the preparations (Table 3). The highest OBC was observed for pea protein (3.24 mL/g), while the lowest was noted for sunflower protein (0.78 mL/g). Compared to other protein sources, the high OBC of pea samples may be attributed to a higher proportion of nonpolar side chains, which facilitate hydrophobic interactions with lipid molecules [6]. In contrast, hemp and sunflower proteins, which contain lower proportions of exposed hydrophobic residues and higher amounts of structural carbohydrates and fiber, exhibited reduced ability to form stable protein–lipid interactions, resulting in lower oil-binding capacity. Pumpkin protein, despite its relatively high fat content, showed intermediate behavior, likely due to competition between endogenous lipids and protein adsorption at the interface. The highest increase in viscosity was observed. The ability of pea ingredients to bind fat allowed them to act as binders, fillers, and functional improvers. Pea protein can mimic some of the functional properties of animal proteins (such as egg whites or dairy) in a wide range of applications, making it versatile for a variety of food product formulations [9]. This is a significant advance in the food industry, especially as companies look for plant-based alternatives to replace animal-based ingredients. Moreover, legume seed proteins have attracted much attention since they could be used in meat products and meat analogues in several forms according to the final product formulation [10]. Similar findings were also noted by other authors, who concluded that the OBC of plant-based protein preparations may be associated with a high fat content [11]. The higher oil-binding capacity of pea protein may be attributed to its higher content of hydrophobic amino acids, which enhances interactions with lipid molecules and contributes to improved fat immobilization and viscosity. In addition, the partial unfolding of protein structures may increase the availability of hydrophobic domains, further enhancing lipid binding. Conversely, proteins with more compact or aggregated structures may exhibit reduced accessibility of such domains, resulting in lower oil-binding capacity.

The color parameters of the protein preparations also varied significantly (Table 3). The highest lightness (L*) was observed for pea protein, whereas hemp protein was characterized by the lowest L* value, indicating a darker appearance. Differences were also noted in the chroma (C) and hue angle (h°), with pumpkin protein showing the highest chroma and a distinct hue compared to other samples. These variations in color may result from differences in the raw material composition and processing conditions. Importantly, the color of the protein preparations may influence the visual properties of the final cream fillings, contributing to the differences observed in Table S1.

### 2.2. Basic Chemical Composition of Cream Fillings

The incorporation of plant-based protein preparations significantly affected the chemical composition of cream fillings (Table S1). In all cases, the addition of protein concentrates resulted in an increase in the protein content compared to the control sample. The protein content increased from 3.64 g/100 g in the control to as much as 7.53 g/100 g in samples with 5% brown rice protein and 7.38 g/100 g with pea protein, which is consistent with the high protein content of these preparations (Table 1).

The protein content of the cream fillings increased in all samples depending on the type and level of protein addition, ranging from 4.77 (2.5% hemp protein) to 7.38 g/100 g (5% pea protein). In contrast, the fat content decreased compared to the control (47.11 g/100 g), with reductions ranging from 2.61 (2.5% pumpkin protein) to 4.80 g/100 g (5% pea protein). The color parameters were also affected by protein addition, with changes observed in lightness (L*), redness (a*), and yellowness (b*) compared to the control.

The lowest fat content was observed in samples with 5% protein addition. This effect can be explained by both the lower fat content of certain protein preparations (Table 1) and their functional properties, particularly the oil-binding capacity (Table 3). Proteins with a higher OBC, such as pea protein, may effectively bind fat within the system, influencing the fat distribution and overall product structure. The fat-absorption capacity of proteins depends on several factors, including the protein source, composition, processing conditions, and structural characteristics [6,12,13], which together determine not only fat distribution within the matrix but also lubrication behavior during oral processing, thereby influencing sensory attributes such as creaminess and melting. Additionally, protein functionality may be affected by the cultivar, extraction method, and molecular composition [10,14,15]. From a mechanistic perspective, fat immobilization in these systems is not only related to absorption but also to the formation of a protein–fat network. Protein aggregates may act as structural fillers within the fat crystal matrix, restricting lipid mobility and contributing to the overall stability of the system. Additionally, competition between proteins and low-molecular-weight surfactants (e.g., lecithin) for interfacial adsorption may further influence the microstructure and distribution of fat droplets.

The color measurement of food products has been used as an indirect measure of other quality attributes such as flavor and contents of pigments because it is simpler, faster, and correlates well with other physicochemical properties [16]. The color of the obtained cream fillings depended on the type and amount of protein preparation used. Based on the results presented in Table 3, it was shown that the lightest cream fillings were the ones with pea protein addition (L* = 78.95, regardless of the amount of added preparation) and with 2.5% addition of brown rice concentrate (L* = 77.32) compared to the control sample (L = 83.45). The darkest were cream fillings with hemp protein addition (L* = 61.11 for 2.5% addition and L* = 54.71 for 5% addition). Most preparations were characterized by a light, pastel color with shades of beige (Table 3). The observed color differences may be related to the presence of naturally occurring compounds in the raw materials and their transformation during processing, which may influence both color and interactions with proteins [17].

One of the factors determining the physicochemical and functional properties of proteins is their diverse amino acid composition [6]. The present study showed that the cream filling differed in terms of amino acid composition (Table 4). Similarly, the observed differences in amino acid compositions of the cream fillings (Table 4) reflected the profiles of the protein preparations used (Table 2). The total amino acid content increased from 35.05 mg/g in the control sample to 72.10 mg/g in fillings with 5% brown rice protein. The highest content of the sum of all amino acids was found in samples with 5% addition of brown rice (72.10 mg/g) and pea protein concentrate (68.21 mg/g), compared with the control sample (35.05 mg/g). Glu was the dominant amino acid in all fillings obtained with protein preparations, which may contribute to enhanced water-binding capacity and influence the viscosity, while the presence of hydrophobic amino acids such as Leu and Ile may promote protein–lipid interactions, thereby affecting fat immobilization and texture perception. Its content ranged from 5.65 mg/g (control sample) to 13.78 mg/g (with brown rice protein). Differences in the content of the amino acid cream fillings resulted from their content in the protein preparations used (Table 1). The content of Arg and Leu in samples with pumpkin and brown rice preparations increased by more than 200%, as compared to a control sample. Pea protein has a well-balanced amino acid profile with a high level of lysine, making it an attractive alternative for enhancing the nutritional quality of plant-based foods [8,18,19,20], which was also confirmed in the present data.

The biological value of the cream fillings depended on the type of protein preparation used. In samples containing pea, hemp, and pumpkin proteins, two limiting amino acids were identified (Phe + Tyr and sulfur amino acids), whereas brown rice and sunflower proteins resulted in a more balanced amino acid profile. Lys was the primary limiting amino acid in most preparations, except for pea protein, where sulfur-containing amino acids were limiting. The amino acid composition of cream fillings reflected the profiles of the applied protein preparations, with the total amino acid content increasing with protein addition, particularly for brown rice and pea proteins. These results indicate that the nutritional value of cream fillings can be effectively modulated through the selection of appropriate plant protein sources. Although PDCAAS was not directly calculated due to the lack of digestibility data, the amino acid profiles suggest that pea and brown rice proteins exhibit higher nutritional quality compared to other sources. The calculation of PDCAAS would require protein digestibility data, which were not determined in the present study. However, based on the amino acid composition, the analyzed protein preparations may be considered valuable sources of dietary protein.

### 2.3. Rheological Measurements of Cream Fillings

The addition of plant-based protein concentrates to cream fillings increased the apparent viscosity of all analyzed samples compared to the control, regardless of the protein concentration (Table 5). This effect can be attributed to the presence of solid components in the system, including milk powder, sugar, and protein preparations, which contribute to the structuring of the continuous phase. Proteins, like other polymers, are known to increase viscosity and enhance the texture and stability of food systems [21]. In contrast, Cheng et al. [8] reported a decrease in viscosity with an increasing soy protein isolate content, which may be related to differences in the composition and structure of the analyzed systems. In the present study, the apparent viscosity increased with a decreasing shear rate, indicating shear-thinning behavior typical of concentrated fat-based dispersions.

The highest increase in viscosity was observed for cream fillings containing a 5% hemp protein preparation, which may be related not only to its higher carbohydrate content but also to the presence of insoluble fractions and fiber, promoting the formation of a more rigid and heterogeneous network compared to more soluble proteins such as pea protein. This behavior contrasts with pea protein, which increased the viscosity primarily through protein–protein interactions and the aggregation of partially unfolded globulins, whereas the hemp-protein-induced viscosity increase was additionally driven by fiber-rich fractions and insoluble carbohydrates, leading to a more heterogeneous and rigid structure. Brown rice and sunflower proteins exhibited weaker structuring effects, consistent with their lower capacity for network formation in the continuous phase. The viscosity at a shear rate of 2 s^−1^ was more than twice that of the control sample. This may be associated with the relatively high carbohydrate content of this formulation compared to the other samples (Table 1). In contrast, cream fillings with pea protein concentrate exhibited viscosity values most similar to the control, regardless of the protein concentration (Table 5). In particular, pea protein was characterized by a higher oil-binding capacity and larger particle size, resulting in increased viscosity but reduced creaminess, whereas hemp and pumpkin proteins contributed to a higher viscosity mainly due to the presence of non-protein components and fiber, leading to a different structural organization and less favorable sensory attributes. The observed behavior can be explained by the fat-mimetic properties of proteins, which are associated with the formation of small aggregates or microgel particles acting as deformable elements within the continuous phase. These structures may enhance viscosity, stabilize dispersed phases, and contribute to the perception of creaminess in reduced-fat systems. This mechanism is often described as the “ball-bearing effect” [4]. However, the efficiency of this mechanism strongly depends on the particle size and deformability. Finely dispersed, deformable protein particles may enhance lubrication, whereas larger, rigid aggregates may increase viscosity but reduce lubrication efficiency, leading to less desirable sensory perception. The observed differences between protein sources may therefore result from variations in aggregation behavior, particle morphology, and the balance between soluble and insoluble protein fractions. Additionally, the oil-binding capacity of protein preparations may contribute to fat immobilization, further influencing the rheological properties and structural organization of the cream fillings. From a molecular perspective, protein functionality in fat-rich systems is governed by hydrophobic interactions between nonpolar amino acid residues and lipid molecules. These interactions promote the formation of structured networks capable of immobilizing fat and stabilizing dispersed phases. The formation of a protein–fat network may contribute to the observed increase in viscosity and stability, acting as a three-dimensional matrix that entraps lipid droplets and solid particles.

It should also be noted that protein denaturation and aggregation are interrelated phenomena. While moderate denaturation may improve functionality by increasing flexibility and interfacial activity, excessive aggregation may lead to the formation of large, insoluble particles with reduced functionality. This balance appears to be critical in determining the performance of protein preparations in cream filling systems.

### 2.4. Particle Size Distribution of Cream Fillings

The particle size distribution is a key parameter influencing both the functional properties and processing behavior of cream fillings. In the present study, the type of protein preparation significantly affected the particle size distribution, whereas the effect of the protein concentration was less pronounced (Table 5). The largest particles were observed in cream fillings containing pea protein (2.90–38.30 µm), compared to the control sample (2.70–27.75 µm). This tendency is specific to legume proteins, where globulin-rich structures promote aggregation under processing conditions. In contrast, rice proteins form smaller and more uniform particles due to their more compact structure, while hemp and pumpkin proteins generate heterogeneous aggregates influenced by fiber and non-protein components, which modify particle-formation pathways during processing. This is consistent with literature data indicating that pea protein particles may range from a few to over 100 µm [22]. The tendency of pea proteins to form larger aggregates may be related to protein–protein interactions and limited solubility under the applied processing conditions, which can be attributed to their specific globulin structure and higher propensity for aggregation compared to proteins from other botanical sources, leading to distinct differences in microstructure and sensory perception. The formation of larger particles may be attributed to enhanced protein–protein interactions driven by hydrophobic interactions and possible disulfide bond formation during processing. The limited solubility of certain protein fractions may further promote aggregation and particle growth.

**Figure 1:**
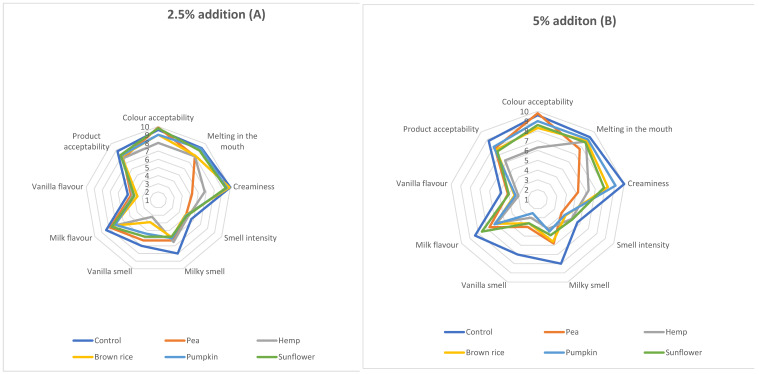
Sensory evaluation of fillings with 2.5% (**A**) and 5% (**B**) selected protein preparations.

In all analyzed samples, the largest fraction of particles corresponded to d90 values, ranging from 30.60 to 38.30 µm. The median particle size (d50) ranged from 9.65 to 11.80 µm for protein-enriched samples, compared to 9.55 µm for the control, with the highest values again observed for pea protein. In contrast, the size of the smallest particles (d10) remained largely unaffected by protein addition. From a technological perspective, particle size plays a critical role in determining both stability and sensory perception. It is generally accepted that particles below approximately 80 µm ensure stable dispersions, whereas larger particles may promote phase separation. However, particles exceeding 25–30 µm may lead to a gritty or sandy mouthfeel, which was also observed in samples containing 5% pea protein in a sensory evaluation of these samples (Figure 1). These findings highlight the importance of controlling protein aggregation during processing. From a formulation perspective, strategies such as particle size reduction, enzymatic modification, or controlled denaturation may help to improve both functional and sensory properties.

The formation of larger protein particles may also influence melting behavior and overall product stability, as reported for protein-based fat replacers in other food systems [23]. At the same time, smaller protein particles, typically in the range of 1–10 µm, are considered more effective in mimicking the textural and lubrication properties of fat. Therefore, although pea protein showed favorable rheological properties, its tendency to form larger particles may negatively affect the sensory quality, indicating a trade-off between technological functionality and mouthfeel. The formation of larger particles may result from limited solubility and enhanced protein–protein interactions under low-moisture conditions, leading to the partial aggregation of protein structures. Additionally, mechanical processing during ball milling may promote the unfolding of protein molecules and subsequent re-association, resulting in the formation of more compact aggregates and an altered particle size distribution. These structural changes may be further influenced by the interfacial properties of proteins, particularly their ability to adsorb and stabilize newly formed surfaces during processing.

Overall, particle size distribution appears to be a key parameter linking molecular-level protein interactions with macroscopic properties such as viscosity, stability, and sensory perception. This highlights the critical role of particle size as a structural parameter that integrates compositional and processing-related effects, ultimately governing both functional performance and sensory perception.

### 2.5. Organoleptic Evaluation of Fillings

These sensory changes may be linked to interactions between protein particles and the fat phase, which influence lubrication, melting behavior, and flavor release during oral processing. Importantly, these interactions differed depending on the protein source. Pea protein particles, due to their higher hydrophobicity and larger size, reduced the lubrication efficiency and increased perceived graininess, whereas hemp and pumpkin proteins influenced sensory perception through structural heterogeneity and the presence of non-protein compounds, affecting flavor release. Rice proteins showed more neutral behavior, resulting in less pronounced sensory deviations from the control. To assess significant differences between fillings with the addition of the analyzed protein concentrates, a sensory descriptive analysis was carried out. Data presented in Figure 1 show the impact of 2.5% and 5% inclusion levels of the protein concentrates on the main sensory descriptors of the obtained fillings. Based on these data, only one out of ten attributes—color homogeneity—showed no significant differences. Significant differences, however, were observed for color acceptability, melting in the mouth, creaminess, milky and vanilla smell, milk flavor, and overall product acceptability.

The first quality parameter evaluated by consumers, critical for product acceptance, was the color of the filling surface. An overview of the subjective color scores for fillings with 2.5% and 5% protein concentrate addition is presented in Figure 1. Regardless of the additive level, fillings with hemp seed protein were scored lowest, appearing grey-green. Other fillings containing pea, brown rice, pumpkin, or sunflower protein concentrates received higher scores, as they were characterized by light beige to grey shades, which were similarly acceptable to the panelists.

**Figure 2:**
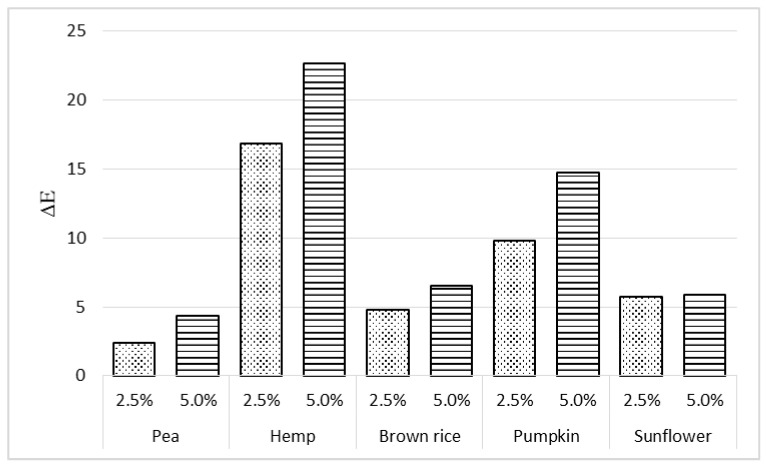
Total color difference (ΔE) of the fillings with 2.5% and 5% addition of selected protein preparations compared to a control sample.

The total color difference (ΔE) is considered one of the most sensitive parameters for quantifying food color changes. Figure 2 shows ΔE values of cream fillings with 2.5% and 5% protein addition compared to the control. The highest color differences were observed for fillings with hemp protein (ΔE = 16.87 and 22.65 for 2.5% and 5%, respectively), consistent with the panelists’ sensory evaluations (Figure 1). According to Adekunte et al. [24], differences in the perceivable color of almost all analyzed samples were classified as perceptibly distinct (ΔE > 3), except for the sample with 2.5% pea protein addition.

Texture and mouthfeel are major determinants of consumer acceptance and preference. The fillings with plant-based protein additions differed in terms of melting in the mouth (Figure 1). The control sample (9.3 points), as well as fillings with 2.5% and 5% pumpkin protein (9.2 and 9.0 points, respectively), exhibited the best melting properties. In contrast, the filling with 5% pea protein showed the lowest melting score (7.7 points), likely due to its lower fat content (42.30 g/100 g), higher protein content (7.36 g/100 g), increased viscosity, and larger particle size, as confirmed by instrumental measurements (Table 5). For hemp and brown rice protein additions, increasing the additive amount improved melting, whereas the opposite trend was observed for pea and sunflower proteins.

Creaminess is another important sensory parameter. Fillings with 2.5% brown rice or sunflower protein, as well as 2.5% and 5% pumpkin protein, showed creaminess similar to the control sample (Figure 1). In general, increasing the protein concentration from 2.5% to 5% decreased creaminess. The exception was the pea protein filling, which exhibited no difference in creaminess between the two inclusion levels and was rated as the least creamy overall. This observation aligns with instrumental measurements of particle diameter (Table 5), indicating the largest particle size in the pea protein filling. Creaminess reflects the interaction of aroma, taste, and texture, and is strongly influenced by microstructural parameters such as the particle size distribution, protein aggregation state, and interfacial behavior, which are in turn governed by protein composition and structural properties. The observed decrease in creaminess and melting properties in samples with larger particle sizes and higher viscosity confirms the strong relationship between microstructure and sensory perception.

Fillings with added pumpkin, hemp, or brown rice protein were rated lowest for milky flavor. This effect may result from the specific aroma and flavor of the added protein concentrates, which can mask the milky and vanilla notes from the base ingredients. Hemp seed contains about 120 aroma-active compounds, mainly volatile terpenes and sesquiterpenes such as α-pinene, myrcene, linalool, limonene, trans-β-ocimene, α-terpinolene, trans-caryophyllene, and α-humulene, contributing to its characteristic aroma. Dominant volatiles in raw pumpkin seeds include lipid aldehydes, ethyl acetate, 2,3-butanedione, and dimethyl sulfide [25], while mature brown rice grains contain mainly aliphatic aldehydes, alcohols, alkanes, and ketones, with minor contributions from aromatic aldehydes, furans, N-heterocyclic compounds, phenols, alkenes, carboxylic acids, and aromatic hydrocarbons (<6%) [26]. These compounds likely masked the inherent milky and vanilla notes of the base filling. Additionally, differences in amino acid composition, particularly in hydrophobic and aromatic residues, may influence flavor perception and interactions with volatile compounds, thereby contributing to differences in sensory attributes between protein sources.

### 2.6. Principal Component Analysis (PCA)

**Figure 3:**
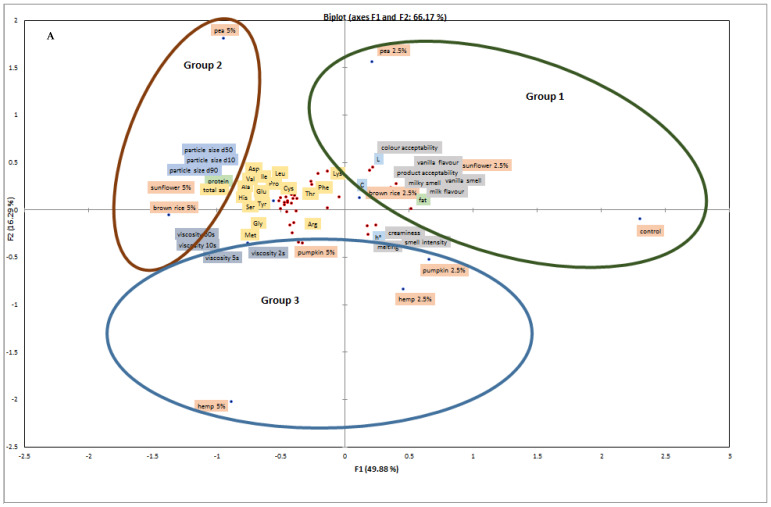

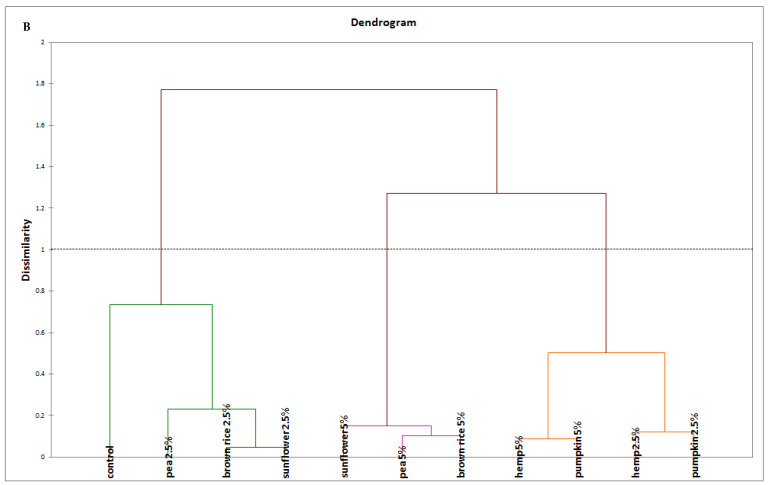
(**A**) Principal component analysis (PCA) and (**B**) cluster maps for fillings with 2.5% and 5% addition of plant-based protein preparations.

PCA was conducted to explore the relationships among physicochemical parameters and sensory attributes in cream fillings with different plant protein preparations (Figure 3A). The first two principal components explained 66.17% of the total variance (PC1 = 49.88%, PC2 = 16.29%).

PC1 primarily captured variation in sensory attributes (color acceptability, vanilla flavor, product acceptability, milky smell, vanilla smell, milk flavor, creaminess, smell intensity, and melting), as well as fat content and color parameters (L, C, h°). PC2 mainly reflected differences in the amino acid composition (Cys, Lys, Tyr, Phe, Ile, Leu, Val, Ser, His, Ala, Gly, Glu, Thr, Met, Pro, Asp, and total aa), viscosity (2–60 s^−1^), and particle size (d10, d50, d90). PCA also revealed that both the type and concentration of the protein preparation affected these multidimensional differences.

The content of individual amino acids (Asp, Thr, Ser, Glu, Gly, Ala, Val, Met, Ile, Leu, Tyr, Phe, His, Arg, and total aa) correlated positively with the total protein content, consistent with previous observations in plant-based protein additions to cookies [27]. L* color parameters correlated positively with sensory descriptors such as color acceptability, vanilla smell, vanilla flavor, product acceptability, and milk flavor. Viscosity showed positive correlations with some amino acids (Thr, Tyr, His, and total aa) and negative correlations with the fat content, L* color, and several sensory attributes (color acceptability, milky smell, vanilla smell, milk flavor, vanilla flavor, product acceptability). Particle size was negatively correlated with melting and creaminess, while other sensory attributes (creaminess, smell intensity, and melting) were negatively correlated with remaining sensory features.

The dendrogram (Figure 3B) grouped the samples into three clusters:

Group 1: control and 2.5% pea, brown rice, and sunflower protein fillings;

Group 2: 5% pea, brown rice, and sunflower protein fillings;

Group 3: hemp protein (2.5% and 5%) and pumpkin protein (2.5% and 5%) fillings.

Overall, PCA confirmed that both the type and concentration of the protein preparation are key factors determining the multidimensional quality profile of cream fillings. The PCA results further confirm that compositional variables (amino acid profile) are directly associated with structural parameters (particle size, viscosity), which in turn are strongly correlated with sensory attributes, supporting the proposed composition–structure–function–sensory relationship.

These multivariate relationships further support the hypothesis that protein functionality in cream fillings is governed by complex interactions among the composition, structure, and aggregation behavior, which collectively determine both physicochemical and sensory properties.

Overall, the obtained results consistently demonstrate that differences between plant protein sources are governed by their specific composition (amino acid profile and non-protein components), which determines structural behavior (denaturation, aggregation, particle size) and consequently translates into functional properties (oil-binding, viscosity, particle size distribution) and sensory perception (creaminess, melting, flavor release). Notably, each protein source exhibited a distinct mechanistic fingerprint: pea protein was primarily governed by hydrophobic aggregation and strong lipid interactions, hemp protein by fiber-driven structural reinforcement, pumpkin protein by lipid–protein-competition effects, brown rice protein by relatively balanced and less interactive structures, and sunflower protein by limited interfacial activity. These source-specific mechanisms explain the observed differences in functional and sensory behavior.

## 3. Materials and Methods

### 3.1. Plant Materials and Plant-Based Protein Preparations

Five commercial plant-based protein concentrates were used: pea (legume), hemp, pumpkin, sunflower (oil seeds), and brown rice (cereal). Proteins were obtained from seeds after cold-pressing oil, followed by drying and fine grinding. Pea and hemp protein preparations were purchased from Bene Vobis (Gdańsk, Poland), and brown rice, pumpkin, and sunflower proteins were from Diet Food (Opatówek, Poland). Detailed chemical composition and amino acid profiles are provided in Table 1 and Table 2. Proteins were incorporated into cream filling formulations at 2.5% and 5% of the total mass. Concentrations were selected based on preliminary technological trials.

The selected protein preparations were chosen to represent different botanical sources (legumes, oilseeds, and cereals), which differ not only in amino acid composition but also in functional properties such as oil-binding capacity, emulsifying ability, and particle-forming behavior. These differences are expected to significantly influence the structure and rheological properties of fat-rich systems. Moreover, all selected proteins are commercially available and increasingly applied in food formulations, which makes them relevant for comparative evaluation in a cream filling system.

Control samples contained no added protein. Experiments were conducted in two independent production batches, and each analytical measurement was performed in triplicate.

### 3.2. Cream Filling Formulation

The base recipe consisted of fractionated palm fat, sugar, milk whey, skimmed milk powder, anhydrous milk fat, and soy lecithin. Selected protein preparations replaced 2.5% or 5% of palm fat. All formulations were processed using ball mill technology. Raw materials were premixed at ~40 °C using a Hobart HL600 floor mixer, then refined in a CAO-B5 laboratory chocolate ball mill (Wormerveer, The Netherlands) for 35 min at 70% maximum agitator speed. Finished fillings were stored in 5 kg containers at ambient temperature until analysis.

### 3.3. Cream Filling Preparation

All recipes were prepared using the ball mill technology, according to the standard recipe. The weighed raw materials were premixed at a temperature of about 40 °C using a Hobart HL600 Floor Mixer equipped with a flat beater (Troy, OH, USA) at level 2. Mixtures prepared in this way were transferred to the CAO-B5 laboratory chocolate ball mill (Wormerveer, The Netherlands). The refining action was accomplished by a special cooled shaft with agitator arms and diverters rotating in a vertical jacketed grinding tank, which was filled with hardened steel balls. The speed of the agitator was set up to 70% of the maximum speed, with a refining time of 35 min. The obtained fillings were stored in 5 kg plastic containers, in a cool, dry place until analysis.

### 3.4. Chemical Composition Analysis

Dry matter, total protein, fat, ash, total and reducing sugars, and crude fiber were analyzed according to AOAC methods [28]. Total nitrogen was determined using the Kjeldahl method [29], fat was determined via Soxhlet extraction, ash was determined via incineration at 550 °C, and sugars were determined using the 3.5-dinitrosalicylic acid method [30]. Total carbohydrates were calculated based on the difference (100—protein—fat—ash—moisture). The values of total sugars and reducing sugars are presented as components of total carbohydrates. Results are expressed as the mean ± SD of three replicates.

### 3.5. Amino Acid Analysis of Protein Preparations and Cream Fillings

The amino acid composition of protein preparations and cream fillings of selected protein preparations was determined via ion-exchange chromatography. Briefly, after 23 h’ hydrolysis with 6 M HCl, the sample was analyzed on the AAA-400 amino acid analyser (INGOS, Prague, Czech Republic), using the ninhydrin method. A photometric detector was used, working at two wavelengths, 440 nm and 570 nm. A column of 350 × 3.7 mm, packed with ion exchanger Ostion LG ANB (INGOS), was utilized. The column temperature was kept at 60–74 °C, with the detector at 121 °C. The calculations were carried out relative to an external standard. The contents of aspartic acid (Asp), threonine (Thr), serine (Ser), glutamic acid (Glu), proline (Pro), glycine (Gly), alanine (Ala), cysteine (Cys), valine (Val), methionine (Met), isoleucine (Ile), leucine (Leu), tyrosine (Tyr), phenylalanine (Phe), histidine (His), lysine (Lys), and arginine (Arg) were determined. No analysis of tryptophan was carried out [31]. Data was reported as the mean value ± standard deviation (SD) for three measurements.

### 3.6. Quantitative Evaluation of Protein Quality

To measure the protein nutritional quality, the amino acid composition of the plant protein preparation was compared with a reference protein. The amino acid pattern for high-quality proteins was established by the Joint Food and Agriculture Organization/World Health Organization Committee in 2007 [32]. Levels were calculated on the basis of the essential amino acid composition of the chemical scores (CSs), according to the Mitchell and Block method [21].

### 3.7. Oil-Binding Capacity of Protein Preparation

The protein suspensions (1 g) were mixed with 5 mL of sunflower oil for 1 min. The samples were then allowed to stand for 30 min. The protein–oil mixtures were centrifuged at 3000× *g* (Rotofix 32A, Hettich, Tuttlingen, Germany), and the unabsorbed oil was carefully collected with a 5 mL calibrated pipette. Oil-binding capacity was expressed in milliliters of oil bound per gram of protein preparation [33]. Data was reported as the mean value ± standard deviation (SD) for three measurements.

### 3.8. Color Analysis

Colour measurements of the fillings were determined at 25 ± 1 °C, using a HunterLab spectrometer D25NC (Birstall, UK), illuminated under standardized conditions (standard light D65, standard Observer CIE 1964/1931 (10°/2°)). Color data are provided as CIE L* (lightness) coordinate, which varies in the range 0–100, as well as chroma (C*) and hue angle (h°) color space parameters calculated from a* (redness-greenness) and b* (yellowness-blueness) values. Chroma (C*) is used to determine the degree of difference of a hue in comparison to a grey color with the same lightness. The higher the chroma value, the higher is the color intensity of samples perceived by humans. Hue angle (h°), considered the qualitative attribute of color, is the attribute according to which colors have been traditionally defined as reddish, greenish, etc., and it is used to define the difference of a certain color with reference to grey color with the same lightness. This attribute is related to the differences in absorbance at different wavelengths. A higher hue angle represents a lesser yellow character in the assays. Chroma and hue values were calculated using the following equations:

(1) $$Hue\;angle\;=\;Arctan\frac{b*}{a*}$$

Chroma = [(a*)^2^ + (b*)^2^]^0.5^ (2)

The instrument was calibrated against a white calibration plate. Each sample was determined in three analytical repetitions, where the read values of L*, a*, and b* were averaged. Total color difference (ΔE) was assessed using a CIE 2000 calculator (Konica Minolta, Osaka, Japan) [34].

### 3.9. Rheological Measurements of Cream Fillings

Rheological properties of cream fillings were measured using a rotational viscometer equipped with a concentric cylinder measuring system—Haake Viscotester iQ (Karlsruhe, Germany) [12]. Viscosity is a critical parameter for technical feasibility. The temperature in the thermostatic bath was set up at 40 °C throughout the viscosity measurement. After the samples were completely melted at 50 °C, they were transferred into the concentric cylinder system of the viscometer and pre-sheared to bring it to equilibrium and to ensure a uniform mass and temperature. Sample viscosity was measured by applying shear rates within the range of 2–60 s^−1^ rate for 10 min at 40 °C. Three analytical repetitions were done to ensure that the differences were not greater than 10%. This analysis was performed at the producer’s (external) laboratory R&D center.

### 3.10. Particle Size Distribution of Cream Fillings

Particle size distribution was measured using the Malvern Mastersizer Micro Chocolate Particle Analyser 3000 (Malvern Instruments Ltd., Malvern, UK), equipped with a small-volume presentation unit MSX1 and an ultrasonic probe. The homogeneous melted samples in quantity 160 ± 5 mg were transferred to the appropriate vessel. Subsequently, 15 mL of Akomed R20 (AAK, Aarhus, Denmark) was added. Then, the samples were agitated continuously during probe sonication to ensure that they were completely dispersed and homogeneous. The prepared samples were analyzed on a Mastersizer device. Read results were provided as a volume frequency (distribution) compared with the particle size curve and characteristic diameters, and parameters for d10, d50, and d90, corresponding to 10%, 50%, and 90% of the volume (mass) of the particle set, were determined. Three analytical repetitions were done for a given test. This analysis was performed at the producer’s (external) laboratory R&D center.

### 3.11. Sensory Evaluation of Cream Fillings

Sensory evaluation of cream fillings was conducted using the quantitative descriptive analysis (QDA) method. The panel consisted of 10 trained assessors (5 females and 5 males, aged 22–55 years) with previous experience in sensory analysis of confectionery products.

Prior to evaluation, the panel underwent refresher training sessions to standardize the perception and interpretation of the evaluated attributes. The attributes included appearance (color intensity and homogeneity), aroma (milky, vanilla, and off-odors), flavor (sweetness, milk flavor, plant-related notes), and texture/mouthfeel (creaminess, melting in the mouth, adhesiveness, and spreadability).

The evaluation was conducted in a sensory laboratory under controlled conditions (temperature 22 ± 1 °C, daylight-type illumination, and individual sensory booths). Samples (approximately 10 g) were served in identical odorless plastic containers coded with random three-digit numbers. The serving order was randomized for each assessor.

Assessors rated the intensity of each attribute using an unstructured 10 cm line scale anchored with the terms “low intensity” and “high intensity”. Between samples, panelists rinsed their mouths with water and consumed unsalted crackers to minimize carry-over effects.

Each sample was evaluated in duplicate in two independent sessions to ensure repeatability of the results.

### 3.12. Statistical Analysis

Analyses of the chemical composition (dry matter, total protein, fat, ash, total and reducing sugars), amino acid profile, viscosity and particle size distribution, oil-binding capacity, and color were performed in three analytical repetitions. Data were subjected to a two-way Duncan’s multiple range test ANOVA (using Statistica 12.0 software). Differences were considered significant at *p* ≤ 0.05. Moreover, a dendrogram analysis (using Euclidean distance based on the Ward method) and principal component analysis (PCA) were carried out. Statistical analyses were performed and SDS values were calculated using Statistica v. 13.0 software (StatSoft Inc., Tulsa, OK, USA). Sensory data were analyzed using analysis of variance (ANOVA) followed by Tukey’s HSD test (*p* < 0.05). Additionally, principal component analysis (PCA) was applied to visualize relationships between sensory attributes and types of protein preparations. The sensory evaluation was conducted with healthy adult volunteers who were informed about the aim and procedure of the study and participated voluntarily. All participants provided informed consent prior to the evaluation. The study followed the ethical principles of the Declaration of Helsinki. According to national regulations, the protocol did not require approval by an ethics committee because no personal or health-related data were collected and the procedure involved only standard food sensory testing.

## 4. Conclusions

The results of this study demonstrate that plant-based protein preparations can be successfully incorporated into cream filling formulations as partial palm fat replacers. Among the analyzed proteins, pea protein showed the most favorable balance between nutritional improvement and technological performance under the studied conditions. It should be emphasized that the observed relationships are specific to the analyzed set of commercial protein preparations and processing conditions. Due to variability in the cultivar, extraction methods, and molecular composition, caution should be exercised when generalizing these findings to other protein systems. The increased protein content (up to ~7.5 g/100 g) indicates the potential for protein-related nutritional claims (e.g., “source of protein”), although final claim eligibility would depend on regulatory criteria. The results also demonstrate that protein functionality in cream fillings is governed by molecular interactions, including hydrophobic interactions, aggregation behavior, and protein–fat network formation, which establish a direct link among the protein composition, structural organization, and the resulting functional and sensory properties of the system.

In general, increasing the protein concentration resulted in higher viscosity and noticeable color changes, while also enabling a reduction in the palm oil content. This is particularly relevant from a sustainability perspective, given the environmental impact associated with palm oil production.

Overall, plant protein preparations represent promising functional ingredients for the development of nutritionally improved confectionery fillings with reduced palm fat contents. Understanding these relationships is essential for the rational design of reduced-fat confectionery systems using plant proteins as functional fat replacers. Future research should focus on optimizing the protein particle size, improving flavor masking strategies, and evaluating storage stability and oxidative changes during prolonged storage.

## Figures and Tables

**Table 1:** Chemical composition of selected plant protein concentrates (g/100 g).

Preparation	Dry Matter	Total Protein	Fat	Ash	Total Sugars	Reducing Sugars	Fiber	Carbohydrates
Pea	93.85 ± 0.03 ^b^	74.90 ± 1.34 ^b^	8.80 ± 0.05 ^c^	4.38 ± 0.04 ^d^	0.51 ± 0.03 ^d^	0.14 ± 0.01 ^c^	0.22 ± 0.07 ^d^	5.77± 0.84 ^d^
Hemp	93.86 ± 0.07 ^b^	45.58 ± 1.13 ^e^	10.51 ± 0.07 ^b^	9.23 ± 0.04 ^b^	3.60 ± 0.02 ^b^	0.29 ± 0.02 ^b^	5.57 ± 0.39 ^a^	28.54 ± 2.47 ^a^
Brown rice	95.52 ± 0.02 ^a^	77.85 ± 1.32 ^a^	5.81 ± 0.09 ^d^	1.65 ± 0.03 ^e^	1.01 ± 0.01 ^d^	0.14 ± 0.03 ^c^	3.30 ± 0.15 ^b^	10.21 ± 1.76 ^c^
Pumpkin	95.43 ± 0.04 ^a^	60.82 ± 0.99 ^c^	12.87 ± 0.06 ^a^	9.65 ± 0.06 ^a^	2.85 ± 0.04 ^c^	0.02 ± 0.02 ^d^	1.78 ± 0.09 ^c^	12.09 ± 1.13 ^c^
Sunflower	92.12 ± 0.05 ^c^	53.57 ± 1.26 ^d^	10.91 ± 0.06 ^b^	6.19 ± 0.02 ^c^	9.43 ± 0.06 ^a^	0.51 ± 0.02 ^a^	3.23 ± 0.32 ^b^	21.45 ± 2.08 ^b^

Values are means ± SDs of three determinations; the same letters (^a–e^) in a column indicates homogenous groups.

**Table 2:** Amino acid composition (mg/g) of selected protein concentrates.

Amino Acid		Protein Preparation				
		Pea	Hemp	Brown Rice	Pumpkin	Sunflower
Asp		15.11 ± 0.65 ^a^	12.69 ± 2.58 ^b^	9.11 ± 0.12 ^c^	7.59 ± 0.20 ^d^	9.47 ± 0.04 ^c^
Thr		4.85 ± 0.18 ^a^	4.11 ± 0.88 ^a^	3.58 ± 0.06 ^b^	2.39 ± 0.08 ^c^	3.03 ± 0.01 ^b^
Ser		6.08 ± 0.31 ^a^	5.70 ± 1.23 ^b^	4.88 ± 0.08 ^c^	3.96 ± 0.14 ^d^	4.25 ± 0.02 ^c^
Glu		20.70 ± 1.13 ^a^	19.99 ± 4.36 ^a^	17.51 ± 0.30 ^b^	14.44 ± 0.47 ^c^	14.71 ± 0.08 ^c^
Pro		5.40 ± 0.28 ^a^	4.35 ± 1.04 ^b^	4.54 ± 0.05 ^b^	2.79 ± 0.21 ^d^	3.48 ± 0.15 ^c^
Gly		5.35 ± 0.11 ^a^	5.27 ± 1.05 ^a^	4.46 ± 0.08 ^b^	4.56 ± 0.11 ^b^	3.86 ± 0.02 ^c^
Ala		6.33 ± 0.12 ^a^	5.75 ± 1.13 ^b^	6.67 ± 0.05 ^a^	4.52 ± 0.08 ^c^	4.29 ± 0.02 ^c^
Cys		0.84 ± 0.01 ^c^	0.99 ± 0.26 ^b^	1.48 ± 0.18 ^a^	0.76 ± 0.05 ^c^	0.76 ± 0.04 ^c^
Val		7.01 ± 0.22 ^a^	6.08 ± 1.30 ^b^	6.44 ± 0.07 ^a^	4.15 ± 0.03 ^c^	4.38 ± 0.00 ^c^
Met		1.45 ± 0.01 ^d^	2.36 ± 0.15 ^b^	2.71 ± 0.03 ^a^	1.54 ± 0.04 ^d^	1.83 ± 0.02 ^c^
Ile		6.37 ± 0.19 ^a^	4.99 ± 1.07 ^b^	4.55 ± 0.04 ^b^	3.37 ± 0.08 ^c^	3.66 ± 0.03 ^c^
Leu		11.22 ± 0.72 ^a^	8.04 ± 1.62 ^b^	9.58 ± 0.07 ^b^	6.03 ± 0.11 ^d^	5.99 ± 0.03 ^d^
Tyr		4.40 ± 0.19 ^b^	3.40 ± 0.74 ^c^	4.97 ± 0.06 ^a^	2.60 ± 0.07 ^d^	3.67 ± 0.04 ^c^
Phe		7.18 ± 0.32 ^a^	5.32 ± 1.04 ^b^	5.72 ± 0.04 ^b^	4.27 ± 0.08 ^c^	4.97 ± 0.05 ^c^
His		3.41 ± 0.10 ^a^	3.32 ± 0.62 ^a^	2.46 ± 0.01 ^b^	2.17 ± 0.06 ^b^	2.51 ± 0.08 ^b^
Lys		10.26 ± 0.81 ^a^	5.14 ± 1.12 ^b^	3.73 ± 0.02 ^c^	3.66 ± 0.12 ^c^	3.84 ± 0.03 ^c^
Arg		11.18 ± 0.63 ^c^	15.19 ± 2.97 ^a^	8.79 ± 0.10 ^d^	13.24 ± 0.35 ^b^	11.38 ± 0.09 ^c^
Ʃaa		127.14 ± 5.96 ^a^	112.69 ± 24.17 ^b^	101.18 ± 1.18 ^c^	82.04 ± 2.36 ^d^	86.08 ± 0.21 ^d^
Limiting amino acid	I	Met + Cys	Lys	Lys	Lys	Lys
	II	-	-	-	Thr	Thr

Values are means ± SDs of three determinations; the same letters (^a–d^) in a column indicates homogenous groups; the bond font means essential amino acids; Ʃaa—sum of all amino acids.

**Table 3:** Oil-binding capacity and color of selected plant protein concentrates.

Preparation	OBC	Color		
	(mL/g)	L*	C	h°
Pea	3.24 ± 0.02 ^a^	84.99 ± 0.07 ^a^	20.33 ± 0.14 ^b^	84.16 ± 0.04 ^d^
Hemp	1.18 ± 0.01 ^c^	57.86 ± 0.09 ^e^	16.46 ± 0.07 ^c^	85.00 ± 0.12 ^c^
Brown rice	2.34 ± 0.01 ^b^	79.25 ± 0.09 ^b^	15.37 ± 0.08 ^d^	80.21 ± 0.19 ^e^
Pumpkin	1.71 ± 0.01 ^c^	74.14 ± 0.04 ^d^	24.60 ± 0.06 ^a^	99.86 ± 0.11 ^a^
Sunflower	0.78 ± 0.01 ^d^	75.10 ± 0.09 ^c^	13.04 ± 0.03 ^e^	86.67 ± 0.04 ^b^

Values are means ± SDs of three determinations; the same letters (^a–e^) in a column indicate homogenous groups; OBC—oil-binding capacity; L*—lightness coordinate varies in the range 0–100; C—chroma; h°—hue angle.

**Table 4:** Amino acid compositions (mg/g) of cream fillings with 2.5% and 5% addition of selected protein concentrates.

Amino Acid		Control	Pea		Hemp		Brown Rice		Pumpkin		Sunflower	
			2.5%	5%	2.5%	5%	2.5%	5%	2.5%	5%	2.5%	5%
ASP		2.84 ± 0.04 ^f^	5.63 ± 0.40 ^d^	7.23 ± 0.07 ^a^	4.62 ± 0.12 ^e^	5.94 ± 0.42 ^d^	3.24 ± 0.19 ^f^	6.66 ± 0.12 ^b^	5.26 ± 0.55 ^d^	6.15 ± 0.23 ^c^	4.14 ± 0.03 ^e^	6.44 ± 0.55 ^c^
THR		1.53 ± 0.34 ^d^	2.56 ± 0.23 ^c^	3.27 ± 0.34 ^a^	2.83 ± 0.14 ^c^	2.88 ± 0.22 ^c^	3.30 ± 0.11 ^b^	3.42 ± 0.10 ^a^	2.30 ± 0.05 ^c^	3.01 ± 0.11 ^b^	2.38 ± 0.01 ^c^	3.48 ± 0.05 ^a^
SER		1.95 ± 0.02 ^e^	2.76 ± 0.28 ^c^	3.62 ± 0.02 ^a^	2.03 ± 0.08 ^d^	3.15 ± 0.27 ^c^	2.59 ± 0.14 ^d^	3.65 ± 0.06 ^a^	3.49 ± 0.41 ^a^	3.37 ± 0.17 ^b^	2.36 ± 0.03 ^d^	3.44 ± 0.37 ^b^
GLU		5.65 ± 0.17 ^f^	10.14 ± 0.78 ^c^	12.81 ± 0.18 ^b^	8.44 ± 0.36 ^d^	10.61 ± 0.78 ^c^	6.59 ± 0.42 ^f^	13.78 ± 0.36 ^a^	7.44 ± 0.53 ^e^	12.61 ± 0.42 ^b^	5.89 ± 0.05 ^f^	8.63 ± 0.53 ^d^
PRO		4.18 ± 0.17 ^e^	5.52 ± 0.15 ^a^	4.97 ± 0.19 ^c^	4.88 ± 0.74 ^c^	4.75 ± 0.17 ^c^	5.19 ± 0.17 ^b^	5.41 ± 0.72 ^a^	5.14 ± 0.42 ^b^	4.65 ± 0.17 ^d^	4.30 ± 0.03 ^d^	4.95 ± 0.42 ^b^
GLY		1.44 ± 0.18 ^d^	1.40 ± 0.10 ^d^	2.06 ± 0.19 ^b^	1.57 ± 0.06 ^d^	1.71 ± 0.12 ^c^	1.51 ± 0.06 ^d^	2.21 ± 0.04 ^a^	1.94 ± 0.39 ^b^	2.18 ± 0.06 ^a^	1.48 ± 0.05 ^d^	1.94 ± 0.41 ^b^
ALA		1.37 ± 0.32 ^f^	2.87 ± 0.18 ^d^	3.19 ± 0.34 ^b^	1.62 ± 0.05 ^f^	2.73 ± 0.16 ^d^	3.57 ± 0.11 ^a^	3.78 ± 0.05 ^a^	2.08 ± 0.25 ^e^	3.07 ± 0.15 ^c^	2.75 ± 0.75 ^d^	3.08 ± 0.23 ^c^
CYS		0.22 ± 0.05 ^e^	0.20 ± 0.03 ^e^	0.26 ± 0.06 ^d^	0.27 ± 0.04 ^d^	0.20 ± 0.03 ^e^	0.60 ± 0.04 ^a^	0.48 ± 0.02 ^b^	0.31 ± 0.07 ^c^	0.31 ± 0.04 ^c^	0.33 ± 0.01 ^c^	0.28 ± 0.07 ^d^
VAL		2.15 ± 0.37 ^f^	3.04 ± 0.26 ^e^	3.97 ± 0.37 ^c^	2.41 ± 0.04 ^f^	3.44 ± 0.25 ^c^	3.63 ± 0.15 ^c^	4.53 ± 0.02 ^a^	3.04 ± 0.15 ^e^	3.69 ± 0.14 ^d^	3.99 ± 0.10 ^c^	4.12 ± 0.22 ^b^
MET		0.56 ± 0.14 ^e^	0.86 ± 0.10 ^d^	1.06 ± 0.13 ^c^	1.15 ± 0.03 ^c^	1.17 ± 0.10 ^d^	1.08 ± 0.02 ^c^	1.72 ± 0.01 ^a^	1.36 ± 0.22 ^b^	1.38 ± 0.02 ^b^	0.63 ± 0.14 ^e^	1.43 ± 0.22 ^b^
ILE		1.95 ± 0.37 ^e^	2.93 ± 0.23 ^d^	3.70 ± 0.34 ^b^	2.06 ± 0.05 ^d^	3.11 ± 0.23 ^c^	3.84 ± 0.07 ^a^	3.83 ± 0.07 ^a^	2.57 ± 0.09 ^d^	3.44 ± 0.09 ^b^	3.43 ± 0.26 ^b^	3.81 ± 0.07 ^a^
LEU		3.38 ± 0.29 ^e^	5.43 ± 0.38 ^c^	6.41 ± 0.29 ^b^	4.57 ± 0.04 ^d^	5.44 ± 0.34 ^c^	5.68 ± 0.15 ^c^	6.82 ± 0.03 ^a^	5.54 ± 0.15 ^c^	5.92 ± 0.18 ^c^	5.57 ± 0.15 ^c^	6.51 ± 0.15 ^a^
TYR		0.16 ± 0.10 ^f^	1.07 ± 0.16 ^d^	1.46 ± 0.13 ^b^	1.17 ± 0.04 ^c^	1.09 ± 0.15 ^d^	0.94 ± 0.08 ^e^	1.82 ± 0.04 ^a^	0.27 ± 0.37 ^f^	1.29 ± 0.06 ^c^	0.58 ± 0.04 ^f^	1.36 ± 0.38 ^b^
PHE		2.45 ± 0.33 ^d^	2.35 ± 0.18 ^d^	3.54 ± 0.34 ^a^	2.37 ± 0.16 ^d^	2.75 ± 0.17 ^c^	2.06 ± 0.11 ^e^	3.66 ± 0.14 ^a^	1.89 ± 0.35 ^f^	3.15 ± 0.14 ^b^	2.47 ± 0.37 ^d^	2.89 ± 0.34 ^b^
HIS		0.36 ± 0.19 ^e^	1.29 ± 0.12 ^c^	1.88 ± 0.17 ^a^	1.47 ± 0.03 ^c^	1.71 ± 0.10 ^b^	1.39 ± 0.08 ^c^	1.95 ± 0.05 ^a^	0.72 ± 0.41 ^d^	1.84 ± 0.08 ^a^	0.94 ± 0.15 ^d^	1.88 ± 0.45 ^a^
LYS		3.45 ± 0.63 ^e^	5.08 ± 0.29 ^b^	5.21 ± 0.61 ^b^	3.68 ± 0.15 ^e^	4.46 ± 0.29 ^d^	5.20 ± 0.14 ^b^	4.81 ± 0.12 ^c^	4.46 ± 0.39 ^d^	4.69 ± 0.16 ^c^	5.54 ± 0.18 ^a^	5.38 ± 0.37 ^a^
ARG		1.41 ± 0.32 ^d^	2.22 ± 0.27 ^c^	3.57 ± 0.33 ^b^	1.95 ± 0.07 ^d^	3.39 ± 0.27 ^b^	2.99 ± 0.04 ^c^	3.57 ± 0.07 ^b^	2.33 ± 0.36 ^c^	4.43 ± 0.08 ^a^	2.25 ± 0.18 ^c^	2.64 ± 0.37 ^c^
Ʃaa		35.05 ± 3.92 ^e^	55.35 ± 4.19 ^c^	68.21 ± 4.12 ^b^	47.09 ± 1.92 ^d^	58.53 ± 4.16 ^c^	53.40 ± 1.99 ^c^	72.10 ± 1.87 ^a^	50.14 ± 1.63 ^d^	65.18 ± 1.99 ^b^	49.03 ± 0.38 ^d^	62.26 ± 1.55 ^b^
Limiting amino acid	I	Phe + Tyr	Met + Cys	Met + Cys	Phe + Tyr	Met + Cys	-	-	Phe + Tyr	Met + Cys	-	-
	II	Met + Cys	Phe + Tyr	-	Met + Cys	-	-	-	Met + Cys	-	-	-

Values are means ± SDs of three determinations; the same letters (^a–f^) in a verse indicates homogenous groups; bond font means essential amino acids; Ʃaa—sum of all amino acids.

**Table 5:** Viscosity and average particle size of cream fillings with 2.5% and 5% addition of selected plant protein concentrates.

Protein Preparation		Control	Pea		Hemp		Brown Rice		Pumpkin		Sunflower	
			2.5%	5%	2.5%	5%	2.5%	5%	2.5%	5%	2.5%	5%
Viscosity [Pa·s]	60 s^−1^	0.80 ± 0.00 ^d^	1.30 ± 0.00 ^b^	1.30 ± 0.00 ^b^	1.30 ± 0.00 ^b^	1.85 ± 0.07 ^a^	1.25 ± 0.07 ^c^	1.95 ± 0.02 ^a^	1.10 ± 0.00 ^c^	1.20 ± 0.00 ^c^	1.10 ± 0.00 ^c^	1.35 ± 0.07 ^b^
	10 s^−1^	1.20 ± 0.00 ^f^	1.85 ± 0.07 ^d^	1.85 ± 0.07 ^d^	2.05 ± 0.07 ^c^	2.80 ± 0.14 ^a^	1.85 ± 0.07 ^d^	2.30 ± 0.14 ^b^	1.60 ± 0.00 ^e^	1.85 ± 0.07 ^d^	1.50 ± 0.00 ^e^	2.05 ± 0.07 ^c^
	5 s^−1^	1.60 ± 0.00 ^e^	2.15 ± 0.07 ^c^	2.15 ± 0.07 ^d^	2.65 ± 0.07 ^a^	3.95 ± 0.21 ^a^	2.35 ± 0.07 ^b^	2.95 ± 0.35 ^b^	2.15 ± 0.07 ^c^	2.50 ± 0.14 ^c^	2.00 ± 0.00 ^d^	2.70 ± 0.14 ^b^
	2 s^−1^	2.45 ± 0.07 ^e^	3.65 ± 0.07 ^d^	3.65 ± 0.07 ^d^	5.15 ± 0.07 ^b^	6.10 ± 0.28 ^a^	3.85 ± 0.21 ^d^	4.40 ± 0.14 ^c^	3.85 ± 0.07 ^d^	4.55 ± 0.21 ^c^	3.40 ± 0.00 ^d^	4.10 ± 0.28 ^c^
Particle size (µm)	*d*10	2.70 ± 0.00 ^b^	2.90 ± 0.00 ^a^	2.90 ± 0.00 ^a^	2.70 ± 0.00 ^b^	2.70 ± 0.00 ^b^	2.70 ± 0.00 ^b^	2.70 ± 0.00 ^b^	2.60 ± 0.00 ^b^	2.70 ± 0.00 ^b^	2.70 ± 0.00 ^b^	2.75 ± 0.07 ^b^
	*d*50	9.55 ± 0.07 ^c^	11.80 ± 0.00 ^a^	11.80 ± 0.00 ^a^	10.40 ± 0.00 ^b^	10.10 ± 0.00 ^b^	10.10 ± 0.00 ^b^	10.20 ± 0.00 ^b^	9.65 ± 0.07 ^c^	10.30 ± 0.00 ^b^	10.05 ± 0.07 ^b^	10.20 ± 0.00 ^b^
	*d*90	27.75 ± 0.21 ^e^	38.30 ± 0.14 ^a^	38.30 ± 0.14 ^a^	34.35 ± 0.07 ^b^	32.0 ± 0.14 ^c^	30.60 ± 0.00 ^d^	32.10 ± 0.00 ^c^	30.95 ± 0.35 ^d^	34.05 ± 0.21 ^b^	31.00 ± 0.28 ^d^	32.25 ± 0.07 ^c^

Values are means ± SDs of three determinations; the same letter in a column (^a–f^) indicates homogenous groups; *d*10 diameter determining 10% of the total amount of particles (μm), *d*50—diameter determining 50% of the total amount of particles (μm), *d*90—diameter determining 90% of the total amount of particles (μm).

## Data Availability

The data are contained within the article.

## Supplementary Materials

The following supporting information can be downloaded at: https://www.mdpi.com/article/10.3390/molecules31101565/s1, Table S1: Chemical compositions and colors of cream fillings with 2.5% and 5% addition of selected plant protein concentrates.
