# Supplementary material for: The Effect of Plant-Based Protein Preparations on Quality and Functional Properties of Cream Filling
Source: Molecules. 2026 May 8;31(10):1565. doi: 10.3390/molecules31101565 (PMC13209638; doi:10.3390/molecules31101565)
Supplement: Supplementary file 1 [file molecules-31-01565-s001.zip › molecules-4240799-supplementary.pdf]

Table S1. Chemical composition and colour of cream fillings with 2.5% and 5% addition of selected plant protein concentrates.

Protein preparation	Addition	Protein (g/100g)	Fat (g/100g)	Colour *L**	Colour C	Colour h°
Control		3.64 ± 0.01[e]	47.11 ± 0.01[a]	83.45 ± 0.16[a]	18.86 ± 0.01[e]	100.89 ± 0.06[b]
Pea	2.5%	5.51 ± 0.01[c]	44.31 ± 0.01[b]	78.96 ± 0.09[b]	22.32 ± 0.03[b]	97.12 ± 0.05[c]
	5%	7.38 ± 0.02[a]	42.31 ± 0.01[c]	78.94 ± 0.06[b]	22.53 ± 0.01[b]	95.28 ± 0.07[d]
Hemp	2.5%	4.77 ± 0.01[d]	44.30 ± 0.00[b]	61.11 ± 0.13[e]	20.51 ± 0.04[c]	98.87 ± 0.03[c]
	5%	5.92 ± 0.01[c]	42.52 ± 0.02[c]	54.71 ± 0.18[f]	18.26 ± 0.01[e]	94.00 ± 0.05[d]
Brown rice	2.5%	5.56 ± 0.01[c]	44.30 ± 0.00[b]	77.32 ± 0.06[b]	19.16 ± 0.05[d]	94.53 ± 0.13[d]
	5%	7.53 ± 0.02[a]	42.52 ± 0.02[c]	75.05 ± 0.07[c]	19.35 ± 0.05[d]	92.90 ± 0.04[e]
Pumpkin	2.5%	5.14 ± 0.01[c]	44.50 ± 0.00[b]	70.61 ± 0.08[d]	24.12 ± 0.01[a]	104.75 ± 0.14[a]
	5%	6.68 ± 0.03[b]	42.51 ± 0.01[c]	64.75 ± 0.13[e]	24.23 ± 0.02[a]	106.03 ± 0.06[a]
Sunflower	2.5%	4.96 ± 0.01[d]	44.30 ± 0.00[b]	75.31 ± 0.13[c]	18.14 ± 0.10[e]	96.83 ± 0.12[c]
	5%	6.33 ± 0.01[b]	43.01 ± 0.01[b]	75.85 ± 0.04[c]	17.12 ± 0.02[f]	96.36 ± 0.07[c]

values are means ±SD of three determinations; the same letter in a column (a-f) indicates homogenous groups; L* (lightness coordinate), varies in the range 0-100; C- chroma; h° (hue angle)
